# Anti-Inflammatory and Gastroprotective Roles of *Rabdosia inflexa* through Downregulation of Pro-Inflammatory Cytokines and MAPK/NF-κB Signaling Pathways

**DOI:** 10.3390/ijms19020584

**Published:** 2018-02-14

**Authors:** Md Rashedunnabi Akanda, In-Shik Kim, Dongchoon Ahn, Hyun-Jin Tae, Hyeon-Hwa Nam, Byung-Kil Choo, Kyunghwa Kim, Byung-Yong Park

**Affiliations:** 1 College of Veterinary Medicine and Bio-safety Research Institute, Chonbuk National University, Iksan 54596, Korea; rashed.mvd@gmail.com (M.R.A.); iskim@jbnu.ac.kr (I.-S.K.); ahndc@jbnu.ac.kr (D.A.); hjtae@jbnu.ac.kr (H.-J.T.); 2 Department of Pharmacology and Toxicology, Sylhet Agricultural University, Sylhet 3100, Bangladesh; 3 Department of Crop Science and Biotechnology, Chonbuk National University, Jeonju 54896, Korea; hh_hh@jbnu.ac.kr (H.-H.N.); bkchoo@jbnu.ac.kr (B.-K.C.); 4 Department of Cardiothoracic Surgery, Research Institute of Clinical Medicine, Chonbuk National University, Jeonju 54907, Korea; tcskim@jbnu.ac.kr

**Keywords:** *Rabdosia inflexa*, inflammation, gastric ulcer, cytokines, MAPK, NF-κB

## Abstract

Globally, gastric ulcer is a vital health hazard for a human. *Rabdosia inflexa* (RI) has been used in traditional medicine for inflammatory diseases. The present study aimed to investigate the protective effect and related molecular mechanism of RI using lipopolysaccharide (LPS)-induced inflammation in RAW 246.7 cells and HCl/EtOH-induced gastric ulcer in mice. We applied 3-(4,5-dimethyl-thiazol-2-yl)-2,5-diphenyltetrazolium bromide (MTT), nitric oxide (NO), reactive oxygen species (ROS), histopathology, malondialdehyde (MDA), quantitative real-time polymerase chain reaction (qPCR), immunohistochemistry (IHC), and Western blot analyses to evaluate the protective role of RI. Study revealed that RI effectively attenuated LPS-promoted NO and ROS production in RAW 246.7 cells. In addition, RI mitigated gastric oxidative stress by inhibiting lipid peroxidation, elevating NO, and decreasing gastric inflammation. RI significantly halted elevated gene expression of pro-inflammatory cytokines such as tumor necrosis factor-α (*TNF-α*), interleukin-1β (*IL-1β*), interleukin-6 (*IL-6*), inducible nitric oxide synthetase (*iNOS*), and cyclooxygenase-2 (*COX-2*) in gastric tissue. Likewise, RI markedly attenuated the mitogen-activated protein kinases (MAPKs) phosphorylation, COX-2 expression, phosphorylation and degradation of inhibitor kappa B (IκBα) and activation of nuclear factor kappa B (NF-κB). Thus, experimental findings suggested that the anti-inflammatory and gastroprotective activities of RI might contribute to regulating pro-inflammatory cytokines and MAPK/NF-κB signaling pathways.

## 1. Introduction

Alcohol consumption is a recognized risk factor for human health. The most common diseases include infectious diseases, gastric ulcer, cancer, diabetes, and liver and pancreas disease caused by alcohol consumption either partially or entirely [1]. The pathogenesis of gastric ulcer is complicated and multifactorial; it is usually caused by an acute imbalance between gastric mucosal integrity and mucosal immunity [2]. Usually, ethanol is absorbed through the intestinal wall and metabolized in the liver in different ways: oxidation by alcohol dehydrogenase (ADH), cytochrome P450 2E1 (CYP2E1), and catalase enzymes. All the processes intensify to form acetaldehyde and then acetate by aldehyde dehydrogenase (ALDH). Alcohol metabolism with ADH enhances the generation of reduced forms of nicotinamide adenine dinucleotide (NADH), but production of CYP2E1 continues to produce free radical. Acetaldehyde and free radicals combine with cell compounds and disturb cell physiology [3]. Consequently, oxidative stress plays a crucial role in the pathogenesis of alcoholic tissue damage and increases lipid peroxidation, which injures capillary endothelial cells and increases cellular permeability [4] that are involved in the DNA damage of gastric mucosal epithelial cells [5]. Although the complete mechanism of alcohol-induced gastric mucosal damage has not been fully disclosed, evidence shows that oxidative stress and neutrophil infiltration are associated with the development of acute gastritis [6,7].

Lipopolysaccharide (LPS), a bacterial endotoxin, is commonly used as an inducer of the macrophage cell lineage, acting through Toll-like receptor 4 (TLR4), which activates the mitogen-activated protein kinases (MAPKs) signaling cascades and the pathway that triggers nuclear factor kappa B (NF-κB) [8,9]. MAPKs are the important signaling pathway and play a crucial regulatory role in both adapted and innate immune response [10]. Ethanol-induced oxidative stress stimulates the release of reactive oxygen species (ROS). ROS are recognized as the second messenger to initiate the redox-sensitive signal-transduction pathway with MAPK cascade and are linked with downstream transcription factor: NF-κB [11]. ROS mediate stimulation of inhibitor kappa B (IκB) kinase, which induces proteasomal breakdown of IκBα and activates NF-κB. NF-κB is a transcription factor that binds to κ-β motifs in the promoters of target genes and triggers transcription of inflammatory cytokines and chemokines [12].

The therapeutic and biological activities of indigenous plants and their active compounds have potential importance for their capability to manage and treat many inflammatory and immunomodulatory diseases [13]. *Rabdosia inflexa* (RI), a perennial shrub, is a member of the lamiaceae family, which is cosmopolitan and cultivated throughout Northeast China, the Korean peninsula, and Japan. In South Korea, RI, locally known as “sanbakha”, has been used as folk medicine for treating gastrointestinal inflammation and pain. Previously, RI and its active compounds such as inflexin and inflexinol have been reported for pancreatitis and anti-cancer effect [14,15,16]. Based on its traditional uses and biological activities, the study investigated its anti-inflammatory and gastroprotective activity and its possible molecular mechanisms in both RAW 264.7 cells and HCl/EtOH-induced gastric ulcer in mice.

## 2. Results

### 2.1. Analysis of Total Phenolic and Flavonoid Contents of Rabdosia inflexa (RI)

Phenolic and flavonoid contents are the secondary metabolites of a plant, having a wide range of biological activities and usually antioxidant properties. The total phenolic and flavonoid content of RI were investigated and presented in Table 1. The total phenolic and flavonoid content of RI were 143.288 ± 1.68 mg/g gallic acid and 256.301 ± 1.40 mg/g rutin equivalent, respectively.

### 2.2. Effect of RI on Viability and Morphology of RAW 264.7 Cells

**Figure 1:**
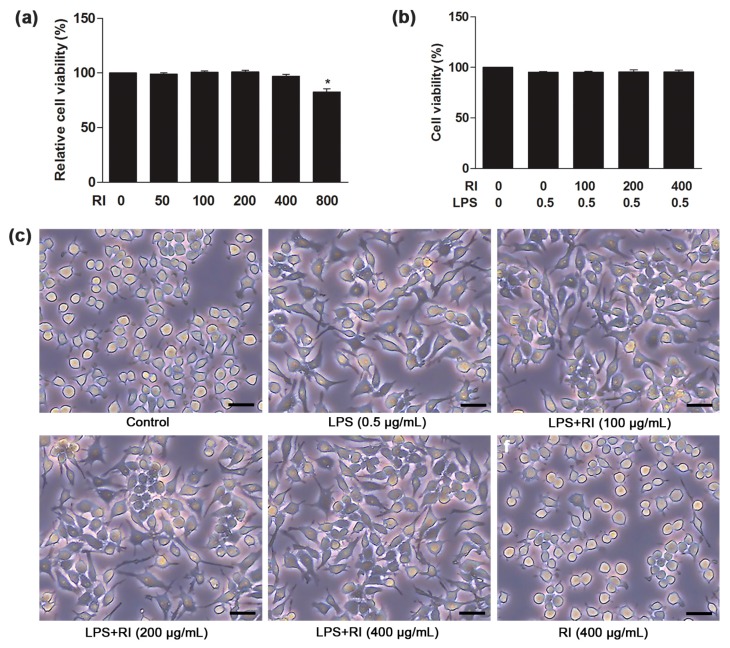
Protective role of *Rabdosia inflexa* (RI) on: (**a**) cytotoxicity; (**b**) cell viability; and (**c**) morphological alteration in RAW 264.7 cells were measured by MTT assay. Cells were pretreated with various concentration of RI (100, 200, and 400 μg/mL) for 1 h, followed by co-treatment with RI and LPS (0.5 μg/mL) for another 24 h. Cell morphology was visualized by optical microscopy (scale bar 200 μm). * *p* < 0.05 when compared with the control. Data are expressed as mean ± SEM of three independent experiments.

The present study measured the anti-inflammatory ability of RI extract using RAW 264.7 cells on LPS-induced inflammation using MTT assay. To investigate the cytotoxicity and cell viability of RI, RAW 264.7 cells were treated with different concentrations of RI (50, 100, 200, 400, and 800 µg/mL) for 24 h. Among the concentrations, RI (800 µg/mL) significantly reduced the cell viability (Figure 1a). However, the cell viability did not significantly alter after co-treatment with LPS (0.5 µg/mL) and RI (100, 200, and 400 µg/mL) for 24 h (Figure 1b). As shown in Figure 1c, LPS markedly induced morphological changes of RAW 264.7 cells after 24 h of treatment, which was consequently improved by the treatment with RI. Thus, results proposed that RI has not affected the viability and morphology of RAW 264.7 cells and it could be due to the anti-inflammatory effect of RI.

### 2.3. RI Attenuated the LPS-Induced NO and ROS Production in RAW 264.7 Cells

**Figure 2:**
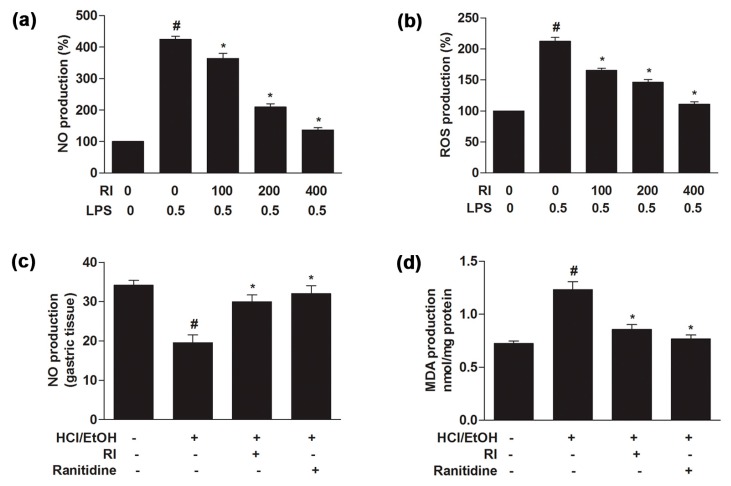
Protective role of RI on NO, intracellular ROS and MDA production in RAW 264.7 cells and gastric tissue. (**a**) NO; and (**b**) ROS production was measured by Griess and ROS-Glo H_2_O_2_ assays in RAW 264.7 cells (upper panel). Cells were pretreated with various concentration of RI (100, 200, and 400 μg/mL) for 1 h, followed by co-treatment with RI and LPS (0.5 μg/mL) for another 24 h. (**c**) NO; and (**d**) MDA production in gastric tissue were measured by Griess and TBARS assays (lower panel). Mice were pretreated for 1 h with RI (400 mg/kg) and Ranitidine (40 mg/kg). After 1 h, HCl/EtOH (10 µL/g) was given orally. # *p* < 0.05 when compared with the control and * *p* < 0.05 when compared with LPS and HCl/EtOH. Data are expressed as mean ± SEM.

In LPS-treated cells, there was a marked increase (*p* < 0.05) in NO and ROS production as compared to the control. Conversely, co-treatment with the RI significantly reduced (*p* < 0.05) the NO and ROS production in a dose-dependent manner (Figure 2a,b). Together, RI suppressed the LPS-induced inflammatory response by preventing NO and intracellular ROS production in RAW 264.7 cells.

### 2.4. RI Improved the Gross and Histopathology of Gastric Tissue

**Figure 3:**
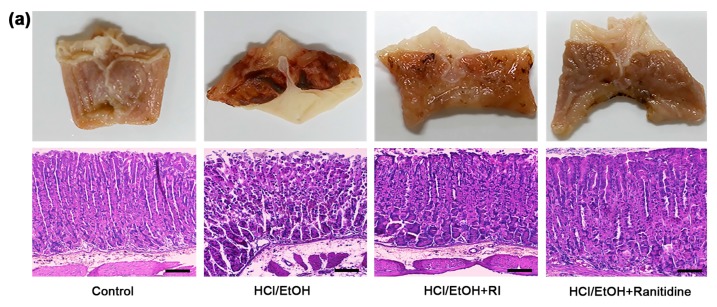

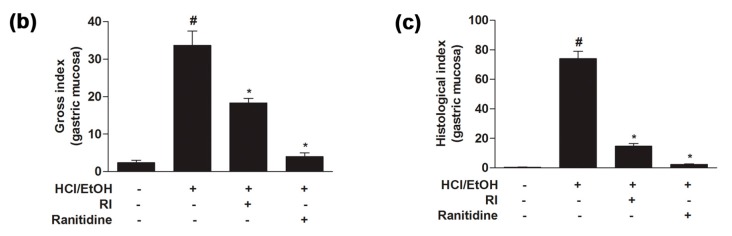
Protective role of RI on HCl/EtOH-induced gastric damage in mice: (**a**) gross lesion (upper panel) and histological lesion (lower panel) (scale bar. 200 μm); (**b**) gross lesion index; and (**c**) histological index. # *p* < 0.05 when compared with the control and * *p* < 0.05 when compared with HCl/EtOH. Data are expressed as mean ± SEM.

The recent study investigated the gastroprotective effect of RI in HCl/EtOH-induced gastric ulcer in mice. HCl/EtOH-induced severe gastric damage, which was notably attenuated by RI pretreatment (Figure 3a, upper panel). In addition, the histological study confirmed that the stomach had the normal structure of mucosa in control group. Besides, in the HCl/EtOH-treated mice, epithelial destruction and inflammatory cells infiltration were found in the mucosa and submucosal area. However, RI and ranitidine-treated groups markedly improved the histopathological changes as compared to HCl/EtOH-treated group (Figure 3a, lower panel). These data are well correlated with the protective abilities of RI against gastric ulcer. Likewise, the gross and histological lesions index of gastric tissue was significantly reduced (*p* < 0.05) by pretreatment with RI and ranitidine treated groups than in HCl/EtOH-induced gastric ulcer mice (Figure 3b,c).

### 2.5. RI Regulated the NO and MDA Production in Gastric Tissue

To evaluate the oxidative stress level, the NO and MDA production was measured in gastric tissue. After inducing gastric injury, HCl/EtOH significantly (*p* < 0.05) decreased NO and increased MDA production. Overall, pretreatment with the RI effectively (*p* < 0.05) increased and decreased the NO and MDA production as related to standard drug ranitidine, respectively (Figure 2c,d). Therefore, results evidently reveal that RI reduced the oxidative stress in HCl/EtOH-stimulated gastric ulcer for its strong anti-oxidant and anti-inflammatory capacity.

### 2.6. RI Suppressed the Activation Pro-Inflammatory Cytokines in Gastric Tissue

**Figure 4:**
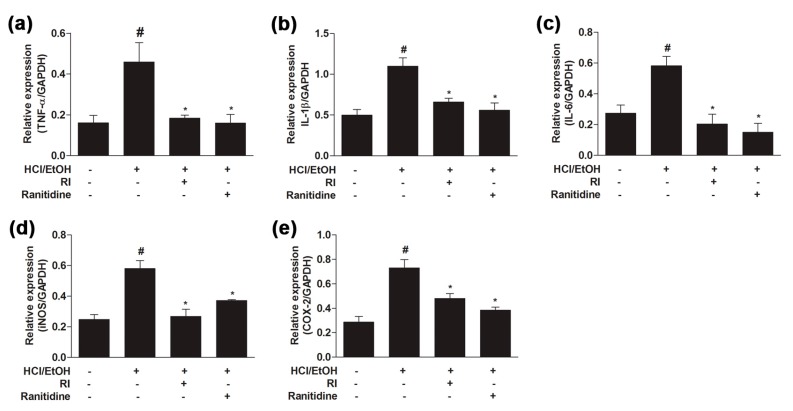
Protective role of RI on gene expression of pro-inflammatory cytokines in gastric tissue. In HCl/EtOH-treated mice, gene expression level of: (**a**) *TNF-α*; (**b**) *IL-1β*; (**c**) *IL-6*; (**d**) *iNOS*; and (**e**) *COX-2* were significantly upregulated, whereas pretreatment with the RI markedly downregulated the gene expression level as related to ranitidine. # *p* < 0.05 when compared with control and * *p* < 0.05 when compared with HCl/EtOH. Data are expressed as mean ± SEM.

Pro-inflammatory cytokines play a fundamental role in various types of inflammation. To elucidate the protective role of RI, the gene expression of pro-inflammatory cytokines was examined in the glandular stomach samples by qPCR analysis. The gene expression level of *TNF-α*, *IL-1β*, *IL-6*, *iNOS*, and *COX-2* were gradually upregulated (*p* < 0.05) in the HCl/EtOH-treated group as compared to the control, whereas pretreatment with RI and ranitidine groups significantly (*p* < 0.05) downregulated the cytokines expression level than in the HCl/EtOH-treated group (Figure 4a–e). Thus, data suggest that RI inhibited the gene expression of pro-inflammatory cytokines in gastric tissue and thereby mitigated the gastric inflammation.

### 2.7. RI Inhibited the COX-2 Expression in Gastric Tissue

**Figure 5:**
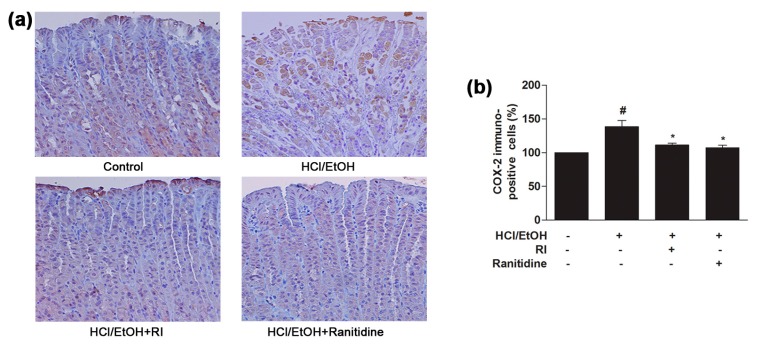
Protective role of RI on COX-2 immunoreactivity in the gastric tissue: (**a**) COX-2 expression in gastric mucosal epithelial cells; and (**b**) COX-2 positive immune-stained cells. Scale bar, 200 μm. # *p* < 0.05 when compared with the control and * *p* < 0.05 when compared with HCl/EtOH. Data are expressed as mean ± SEM.

It is recognized that elevated expression of COX-2 plays a vital role in the inflammatory process and previous study has revealed that HCl/EtOH strongly activates COX-2 expression in gastric tissue [17]. COX-2 expression in the gastric mucosal epithelial cells was revealed by immunohistochemical staining analysis. As observed, COX-2 was slightly expressed in the normal control gastric mucosal epithelial cells; in contrast, HCl/EtOH increased the COX-2 expression of gastric mucosal epithelial cells, which was mostly observed in the gastric mucosal inflammatory area (Figure 5a). The expression of COX-2 was markedly (*p* < 0.05) blocked by the pretreatment of RI as related to the standard drug ranitidine (Figure 5b). Thus, RI significantly blocked the activation of COX-2 expression in the gastric mucosal inflammatory area and reduced the inflammatory activity.

### 2.8. RI Blocked the MAPK Cascade, COX-2, and NF-κB Activation

**Figure 6:**
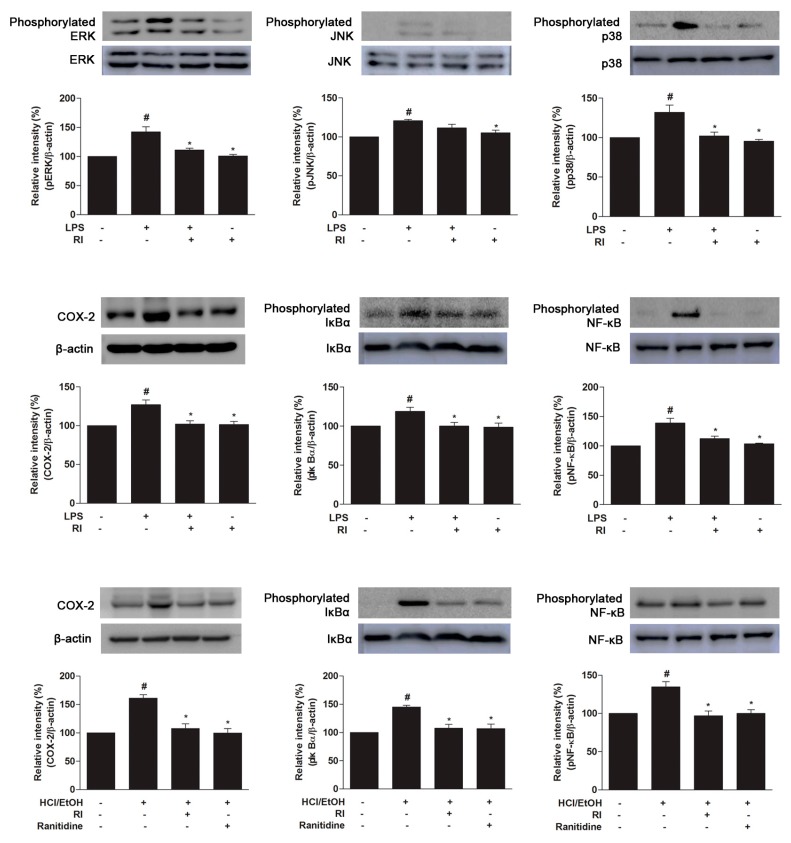
Protective role of RI on the MAPK cascades, COX-2 expression, and activation of IκBα, NF-κB in RAW 264.7 cells and gastric tissue. Here, upper and middle panels represent the MAPKs (pERK1/2, pJNK, and pp38), COX-2, IκBα and NF-κB expression in RAW 264.7 cells and the lower panel represents the COX-2, IκBα and NF-κB expression in the gastric tissue. The relative band intensity of target protein was measured as compared with total protein and β-actin. LPS-induced the phosphorylation of MAPK cascade, whereas pretreatment with the RI reduced the phosphorylation of MAPK cascade. LPS and HCl/EtOH increased the COX-2 expression, kinetic phosphorylation, and degradation of IκBα and phosphorylation of NF-κB. However, pretreatment with the RI notably decreased the COX-2 expression, IκBα phosphorylation, and degradation, NF-κB translocation as related to standard drug ranitidine. # *p* < 0.05 when compared with the control and * *p* < 0.05 when compared with LPS and HCl/EtOH. Data are expressed as mean ± SEM.

To find the possible molecular mechanisms of the anti-inflammatory and gastroprotective role of RI, the protein expression related to anti-inflammation signaling pathways was evaluated. The present data showed that LPS treatment remarkably elevated the phosphorylation of MAPK family protein (ERK1/2, JNK, and p38) in RAW 264.7 cells, whereas RI pretreatment notably (*p* < 0.05) attenuated the phosphorylation of MAPK proteins (Figure 6, upper panel). Meanwhile, LPS and HCl/EtOH treatment increased COX-2 expression in RAW 264.7 cells and gastric tissues were markedly (*p* < 0.05) blocked by RI pretreatment (Figure 6, middle panel). After LPS and HCl/EtOH stimulation, IκBα and NF-κB phosphorylation were noticeably (*p* < 0.05) increased, indicating the activation of NF-κB. However, IκBα phosphorylation and the nuclear translocation of NF-κB (p65) were gradually reduced (*p* < 0.05) by RI pretreatment (Figure 6, middle and lower panels). Moreover, RI alone does not seem to involve in the signal pathways in vitro study. Together, these results demonstrate that RI significantly inhibited the phosphorylation of MAPK cascade in RAW 264.7 cells as well as activation of COX-2, IκBα, and NF-κB in RAW 264.7 cells and gastric tissues, simultaneously.

## 3. Discussion

Gastrointestinal disorders are a global health problem affecting millions of people. Inflammation is a defensive biological response to harmful stimuli and infection that promotes the production of inflammatory mediators. Oxidative stress plays a vital role in alterations related to the pathophysiology of inflammation. Modulation of the inflammatory mediators is considered a promising strategy for facing inflammatory disease. Although RI has been traditionally used to treat inflammatory diseases, the underlying molecular mechanism of anti-inflammation properties is still not understood. The study investigated the anti-inflammatory and gastroprotective activity of RI in a model of LPS-induced inflammation in RAW 264.7 cells and HCl/EtOH-induced experimental gastric ulcer.

Antioxidants play an important role in redox mechanisms in a biological system, protecting it against inflammation and apoptosis. Phenolic and flavonoid are the most important plant secondary metabolites and have the strong antioxidant capacity [18,19]. Antioxidants act as oxygen scavenger capable of catalyzing the oxidative process [20]. Significant amounts of phenolic and flavonoid content were found in RI (Table 2) that may be the major donor for the anti-oxidative as well as anti-inflammatory role against gastric damage. For the possible mechanism by which RI protects against LPS-induced macrophage cell damage, these results showed that it may act through its anti-oxidative and anti-inflammatory effects (Figure 1). Treatment with the RI inhibited the LPS-induced intracellular oxidative stress (ROS) and NO production (Figure 2, upper panel). These data strongly suggest that RI could cure various inflammatory symptoms based on its anti-inflammatory properties. Tissue-related macrophages play an important role in the loss of physiological functions of the organ by releasing toxic and inflammatory molecules, such as NO and ROS [21]. The synthesis of these inflammatory molecules is responsible for the progression of various inflammatory diseases, such as gastric ulcer [22,23].

The pathogenesis of ethanol-induced gastric injury is very complex and related to oxidative stress that has been confirmed by recent studies [24,25]. Gastric tissue damage is caused by an imbalance between the damage of the gastric tissue and protective factors. In addition, NO plays a complex role in gastric mucosal integrity and its synthesized independently [26]. NO level declines in patients suffering from gastric distress considerably. By increasing gastric mucosal blood flow, normal production of NO could retain the integrity of gastric mucosa and contribute to the defense and healing of mucosal damage, while preventing chemotaxis and adhesion of inflammatory cells to guard gastric mucosa [27]. Following gastric damage, the gastric tissue may be partially oxidized due to injury. Lipid peroxidation is the result of ROS reaction against cell membranes and produces a significant level of pro-oxidant such as MDA, which leads to oxidative gastric damage [28]. In the present study, RI markedly increased NO and decreased MDA levels in gastric mucosa, demonstrating the anti-inflammatory and antioxidant potential of RI (Figure 2, lower panel). This finding is consistent with an earlier report [27]. In this study, RI attenuated the macroscopic and histopathologic lesions in gastric mucosa and inflammatory cells influx that signifies its prospective anti-gastric ulcer activities (Figure 3), as also shown for the standard compound ranitidine hydrochloride, a histamine-2 receptor antagonist clinically recommended for the treatment of gastric ulcer [29]. The ethanol-induced gastric lesion is a key experimental model commonly used for likely anti-gastric ulcer activity since ethanol is thought to be a leading cause of gastric ulcer [30,31]. Ethanol has been revealed to cause hemorrhagic gastric lesions characterized by mucosal friability and infiltration of inflammatory cells [32]. Reduction of the infiltration of inflammatory cells (neutrophil) has been considered to be a vital anti-inflammatory mechanism by which effective anti-gastric-ulcer medicine protects against mucosal injuries [33]. 

Pro-inflammatory cytokines and enzymes such as *TNF-α*, *IL-1β*, *IL-6*, *iNOS* and *COX-2* genes in the tissue may be used as biomarkers of gastric visceral damage. Following inflammatory stimuli, inflammatory mediators have been elevated to prompt deleterious effect in the stomach. In this study, increased production of inflammatory cytokines in ulcerated gastric tissue can be attributed to the damaging effect of ethanol. A high level of cytokines triggers neutrophils, lymphocytes, and monocytes at the inflammatory site; these, in turn, start a different oxidative disturbance, toxic metabolites, and lysosomal enzymes liable for local tissue damage in gastric ulcer [34]. Present study observed that RI remarkably suppressed the pro-inflammatory cytokine production in gastric tissue (Figure 4). The inhibition of NF-κB is a primary mechanism for RI suppression of gastric ulcer since the expression of various pro-inflammatory cytokines including *TNF-α* is mainly regulated by the transcription of NF-κB [35].

Inflammation is a physiological and immunological response triggered by both cell and tissue injury that is primarily controlled by a MAPK cascade signaling pathway [36]. MAPKs are kinases that are responsible for most cellular responses to inflammatory cytokines and external stress signals, and these kinases are essential for the regulation of the production of various inflammation mediators [37,38]. MAPK cascade pathway is activated by inflammatory stimuli such as LPS. MAPK comprising ERK, JNK, and p38, is controlled in response to the triggering of extracellular signal cytoskeletal proteins, nuclear transcription factors and the stabilization of cytokines gene. Experimental results revealed that LPS gradually upregulated the phosphorylation of MAPKs, and these phosphorylated MAPKs was notably inhibited by pretreatment with RI, resulting in the elevation of antioxidant response element of RI regulated phase II enzymes, which are involved in cellular protection mechanism (Figure 6, upper panel). Similarly, COX-2 is an important factor which plays key roles in the pathogenesis of inflammation. The protein expression of COX-2 significantly increased after LPS and HCl/EtOH treatment. However, the expression of COX-2 was decreased markedly by RI treatment (Figure 6, middle and lower panels). The immunohistochemical data showed that RI pretreatment markedly blocked the COX-2 localization in the gastric mucosal epithelium cells of the inflammatory area indicating that COX-2 was involved in the inhibition of inflammatory cells activation and mitigates the oxidative stress, and improve the healing process in the gastric mucosa (Figure 5).

It is known that pro-inflammatory cytokines are regulated by NF-κB signaling pathway [39]. The activation of NF-κB is induced by ROS and NO produced from macrophages following exposure of LPS and it depends on the phosphorylation and degradation of the corresponding upstream factor IκBα. Similarly, gastric damages lead to the production of free radicals that prompt the migration and accumulation of macrophages and leukocytes in the damaged sites and the release of pro-inflammatory mediators [40]. The NF-κB dimers are normally sequestered in the cytosol by binding to the IκB inhibitory protein. The previous study reported that ethanol stimulation activates NF-κB, which leads to phosphorylation of IκBα and p50/p65 heterodimer [27]. These p50/p65 dimers enhanced by phosphorylated IκBα promote gene expression of pro-inflammatory cytokines, which translocate to the nucleus [41]. In the present study, the results showed that RI significantly inhibited phosphorylation of IκBα and NF-κB p65 in RAW 264.7 cells and gastric tissue after LPS and HCl/EtOH stimulation, respectively (Figure 6, middle and lower panels). The study indicates that RI may inhibit early steps of inflammation and modulate upregulation of pro-inflammatory cytokines through suppression of NF-κB translocation. 

## 4. Materials and Methods

### 4.1. Chemicals and Antibodies

LPS, MTT, penicillin/streptomycin, trypsin-EDTA, hematoxylin, eosin, and protease inhibitor were purchased from Sigma-Aldrich (St. Louis, MO, USA). DMEM, FBS, and other cell culture reagents were supplied by Gibco (Carlsbad, CA, USA). DMSO was obtained from Bioshop (Burlington, Ontario, Canada). RNA extraction kit (RiboEx and Hybrid-R) was bought from Gene All (Seoul, Korea). Griess reagent, cDNA synthesis kit (ReverTra Ace qPCR RT Kit), T-PER, and BCA protein assay kit were purchased from Thermo Scientific (Waltham, MA, USA). SYBR Green qPCR Kit obtained from TOYOBO (Tokyo, Japan). Primary antibodies ERK1/2, JNK, p38, COX-2, IκBα, NF-κB, and β-actin were supplied by Cell Signaling (Danvers, MA, USA). Secondary antibody (goat anti-rabbit immunoglobulin g horseradish peroxidase) was provided by Santa Cruz (Dallas, TX, USA). WESTSAVE gold ECL detection kit was obtained from Abfrontire (Seoul, Korea). TBARS assay kit by Cayman (Ann Arbor, MI, USA). ROS-Glo H_2_O_2_ assay kit was supplied by Promega (Madison, WI, USA). Zoletil 50 was bought from Virbac (Carros, France).

### 4.2. Collection and Preparation of Rabdosia Inflexa Extract

The aerial part of RI was collected from the Jiri mountain area in the southern part of Korea and was authenticated from its microscopic and macroscopic features by the Korean Institute of Oriental Medicine (KIOM). We prepared RI extract according to the previously described method [42]. Briefly, the aerial parts were chopped and dried completely. The extract was prepared by maceration of the sample with 70% ethanol (twice for 2 h reflux), and then the filtered extract was concentrated under vacuum centrifuge and dehydrated with a lyophilizer. The powder extract was liquefied in dimethyl sulfoxide (DMSO) and was sterilized using a 0.22 μm syringe filter. RI was dissolved in 0.04% DMSO in media for cell culture experiments and 0.1% DMSO in saline for oral gavage. The dried extract was kept at −20 °C. The study was conducted using a single batch of extract to avoid batch-to-batch variation and maximize the product constancy.

### 4.3. Phytochemical Analysis

Total phenolic and flavonoid content of RI extract was measured using Folin–Ciocalteu (FC) method according to the previously described method [43].

### 4.4. RAW 264.7 Cells Culture

Mouse macrophage RAW 264.7 cells were cultured in Dulbecco modified Eagle medium (DMEM) enriched with 10% fetal bovine serum (FBS) and 1% penicillin and streptomycin in a 5% CO_2_ humidified incubator at 37 °C. Cells were maintained as a monolayer and subcultured once cells reached about 90% confluency in the culture flask.

### 4.5. Cell Viability and Morphological Study

Cell viability was detected using a 3-(4,5-dimethyl-thiazol-2-yl)-2,5-diphenyltetrazolium bromide (MTT) assay, following a prescribed method [34]. Briefly, RAW 264.7 cells (1 × 10^6^ cells/mL) were cultured overnight. To determine the cytotoxicity, cells were treated with RI (50, 100, 200, 400, and 800 µg/mL) for 24 h. In contrast, measuring the cell viability, cells were pretreated with RI (100, 200, and 400 µg/mL) for 1 h and co-treated with LPS (0.5 µg/mL) and RI for another 24 h. Moreover, for morphological evaluation of RAW 264.7 cells, the image of the cells was acquired by an inverted microscope (CKX41, Olympus, Tokyo, Japan). 

### 4.6. Measurement of NO and ROS in RAW 264.7 Cells

Mouse macrophage RAW 264.7 cells (1 × 10^6^ cells/mL) were cultured in 96 well plates. After overnight culture, cells were pretreated with RI (100, 200. and 400 µg/mL) for 1 h and were then co-treated with LPS (0.5 µg/mL) and RI for 24 h. NO, and ROS activity was measured by Griess reagent and a ROS-Glo H_2_O_2_ assay kit according to the manufacturer’s recommendation. Intracellular ROS and NO levels were measured at 570 nm by the tunable Versa max microplate reader.

### 4.7. Mice Management and HCl/EtOH-Induced Gastric Ulcer Model

Six-week-old ICR mice were handled in accordance with the published method [34] and in accordance with the guide for the care and use of laboratory animals (Eighth Edition, 2011, published by The National Academies Press, Washington, DC, USA) and Institutional Animal Care and Use Committee (IACUC; CBNU 2017-0126) of the Chonbuk National University Laboratory Animal Center in Korea. An experimental gastric ulcer model in mice was induced using HCl/EtOH [44]. Forty ICR mice were randomly divided into four groups. Mice fasted overnight before the experiment. Fasted mice were orally given RI (400 mg/kg) and ranitidine (40 mg/kg) in respected groups for 1 h. After 1 h, 60% EtOH in 150 mM HCl (10 µL/g) was given orally. Control mice were treated with normal saline. Mice were anesthetized with Zoletil 50 (10 mg/kg) 1 h after administration of HCl/EtOH and samples were collected for experimental analysis.

### 4.8. Gross and Histopathology of Gastric Mucosal Tissue

To evaluate the gross and histological changes in glandular stomach tissue, we followed previous study [34]. For quantifying the degree of gross and histological lesions index, we followed a prescribed method with slight modification [45]. Briefly, the gross lesions of gastric mucosa were measured using following formula; (number of lesions of type I) + (number of lesions of type II) × 2 + (number of lesions of type III) × 3. Here, the gross lesions were characterized by: the presence of single submucosal punctiform hemorrhage, edema, type I; the presence of submucosal hemorrhagic lesions with slight erosions, type II; and the presence of deep ulcer with erosions and invasive lesions, type III. Besides, the histological lesions of gastric mucosa were measured using following formula; (% type I lesion) × 1 + (% type II lesion) × 2 + (% type III lesion) × 3. Here, the histological lesions were characterized by: gastric mucosal cells appeared intact and had a normal shape, type 0 lesion; surface epithelial cells and the uppermost 2 or 3 cells lining the glands were damaged, type I damage; damage greater than I but involving <50% of the thickness of the gastric mucosa, type II damage; and damage involving >50% of the thickness of the gastric mucosa, type III damage.

### 4.9. Analysis of Lipid Peroxidation and NO Production

Malondialdehyde (MDA) and NO are important indicators of oxidative stress. The gastric tissue was homogenized and centrifuged at 10,000 rpm at 4 °C for 10 min. The supernatant was collected and kept at −80 °C for experimental analysis. MDA and NO concentration were measured in the gastric tissue samples according to the commercial kit instructions.

### 4.10. RNA Extraction and Quantitative Real-Time Polymerase Chain Reaction (qPCR)

The RNA was extracted from a gastric tissue according to the manufacturer’s instructions. The concentration of total RNA was quantified with the BioSpec-nano spectrophotometer (Shimadzu Biotech, Tokyo, Japan) at a 260/280 nm ratio. For complementary DNA (cDNA) synthesis, total RNA (3 μg) was used, and cDNA synthesis was maintained according to the manufacturer’s instructions. qPCR was performed SYBR Green Real-Time PCR master mix according to Roche LightCycler™. Relative expression of target genes was normalized to the reference gene: glyceraldehyde 3-phosphate dehydrogenase (GAPDH). The sequences of the primers (Bioneer, Daejeon, Korea) used are shown in Table 2 [46].

### 4.11. Immunohistochemical (IHC) Analysis

COX-2 immunopositive cells expression in the gastric tissue was performed using according to Vectastain ABC kit recommendations. Briefly, paraffin section was deparaffinized in xylene and hydrated in ethanol. Citrate buffer was used for antigen retrieval and 3% hydrogen peroxide (H_2_O_2_) was used for inactivating the endogenous peroxidase activity. Tissue was blocked with normal serum for 1 h. Anti-rabbit monoclonal COX-2 antibody (dilution 1:200) was incubated overnight at 4 °C. Subsequently, the section was incubated with biotinylated secondary antibody for 1 h and Vectastain ABC reagent for 30 min at room temperature. The sections were incubated with diaminobenzidine (DAB) in the dark until brown color development. After counterstain, the section was dehydrated in ethanol, cleared in xylene and mounted on a glass slide. The section was imaged at a fixed 100× magnification using Leica DM2500 microscope (Leica Microsystems, Wetzlar, Germany).

### 4.12. Western Blot Analysis

RAW 264.7 cells and gastric tissue were harvested and washed twice with ice-cold PBS. Cells and tissues were lysed by the lysis buffer; radioimmunoprecipitation assay buffer (RIPA), and/or tissue protein extraction reagent (T-PER), phenylmethanesulfonyl fluoride (PMSF), sodium orthovanadate (Na_3_VO_4_), and protease inhibitor cocktail. The total concentration of protein of lysate cells and tissues were measured with a bicinchoninic acid (BCA) protein assay protein kit. An equal amount of protein was separated by 10–12% sodium dodecyl sulfate-polyacrylamide gel electrophoresis (SDS-PAGE) and transferred to a nitrocellulose membrane. The membrane was incubated with blocking serum; 5% bovine serum albumin (BSA) in Tris-buffered saline with tween twenty (TBST) for 2 h at room temperature and by primary antibodies for overnight at 4 °C. Then, the blot was washed and incubated with secondary antibodies for 2 h. Bands were detected using an enhanced chemiluminescence (ECL) detection kit, and bands images were taken by a LAS-400 image system, (GE Healthcare, Little Chalfont, UK); β-actin was used as the reference antibody.

### 4.13. Statistical Analysis

Data were analyzed with Graph Pad Prism 5.0 (Graph Pad Software, Inc., San Diego, CA, USA) and are expressed as mean ± standard error (SEM). Statistical analyses were assessed by analysis of variance (ANOVA) followed by Bonferroni post-hoc tests. The minimum statistical significance was considered to be *p <* 0.05 for all analyses. 

## 5. Conclusions

RI mitigates inflammation and maintains normal gastric mucosal integrity. The present study validates that RI protects against inflammation and gastric ulcer by mitigating the inflammation response and oxidative stress via downregulation of the pro-inflammatory cytokines mediated by MAPK/NF-κB signaling pathways. Therefore, RI could be a promising phytomedicine and has advantages for prospective clinical applications in the future for an oxidative stress-mediated gastric ulcer.

## Figures and Tables

**Table 1:** Total phenolic and flavonoid content of *Rabdosia inflexa* (RI).

Plant Extract	Total Phenolic (mg GAE/g Extract)	Total Flavonoid (mg RU/g Extract)	Total Yield (%)
RI	143.288 ± 1.68	256.301 ± 1.40	27.13

Note: Gallic acid and rutin were used as standards. Results are expressed in milligrams of gallic acid equivalent per gram of extract sample (mg GAE/g) and mg of rutin equivalent per gram of extract sample (mg RU/g).

**Table 2:** The nucleotide sequence of the primers for qPCR.

Gene	Primers Sequence (5′–3′)	Genebank Accession No.
*TNF-α*	TTGACCTCAGCGCTGAGTTG	NM_013693
	CCTGTAGCCCACGTCGTAGC	
*IL-1β*	CAGGATGAGGACATGAGCACC	XM_006498795
	CTCTGCAGACTCAAACTCCAC	
*IL-6*	GTACTCCAGAAGACCAGAGG	NM_001314054
	TGCTGGTGACAACCACGGCC	
*iNOS*	CCCTTCCGAAGTTTCTGGCAGCAGC	XM_006532446
	GGCTGTCAGAGCCTCGTGGCTTTGG	
*COX-2*	CACTACATCCTGACCCACTT	NM_011198
	ATGCTCCTGCTTGAGTATGT	
*GAPDH*	CACTCACGGCAAATTCAACGGCAC	XM_017321385
	GACTCCACGACATACTCAGCAC	
