# Correction: Akanda, M.R., et al., Anti-Inflammatory and Gastroprotective Roles of *Rabdosia inflexa* through Downregulation of Pro-Inflammatory Cytokines and MAPK/NF-κB Signaling Pathways. *Int. J. Mol. Sci.* 2018, *19*, 584

**DOI:** 10.3390/ijms20061495

**Published:** 2019-03-25

**Authors:** Md Rashedunnabi Akanda, In-Shik Kim, Dongchoon Ahn, Hyun-Jin Tae, Hyeon-Hwa Nam, Byung-Kil Choo, Kyunghwa Kim, Byung-Yong Park

**Affiliations:** 1 College of Veterinary Medicine and Bio-safety Research Institute, Chonbuk National University, Iksan 54596, Korea; rashed.mvd@gmail.com (M.R.A.); iskim@jbnu.ac.kr (I.-S.K.); ahndc@jbnu.ac.kr (D.A.); hjtae@jbnu.ac.kr (H.-J.T.); 2 Department of Pharmacology and Toxicology, Sylhet Agricultural University, Sylhet 3100, Bangladesh; 3 Department of Crop Science and Biotechnology, Chonbuk National University, Jeonju 54896, Korea; hh_hh@jbnu.ac.kr (H.-H.N.); bkchoo@jbnu.ac.kr (B.-K.C.); 4 Department of Cardiothoracic Surgery, Research Institute of Clinical Medicine, Chonbuk National University, Jeonju 54907, Korea; tcskim@jbnu.ac.kr

The authors wish to make the following corrections to this paper [1]: 

There were some mistakes in Figure 6 of the original version of the published paper (page 7). The authors have changed the total form bands of IκBα, and NF-κB. Unfortunately, in the middle panel, the authors have used a total form of the IκBα protein band in the reverse direction, and also in the lower panel, the same bands of the total form of in vitro and in vivo IκBα and NF-κB proteins were used. Therefore, the authors have corrected the errors as shown in Figure 6. The authors have modified the total form of the in vitro IκBα band (middle panel) and also replaced the total form bands of in vivo IκBα and NF-κB proteins (lower panel). The sentence in the figure’s legend “The relative band intensity of target protein was measured as compared with total protein and β-actin” should be corrected to “The relative band intensity of target protein was measured as compared with β-actin”. The other parts of the manuscript do not need to be changed.

Figure 6 should be replaced with the following:

**Figure 6:**
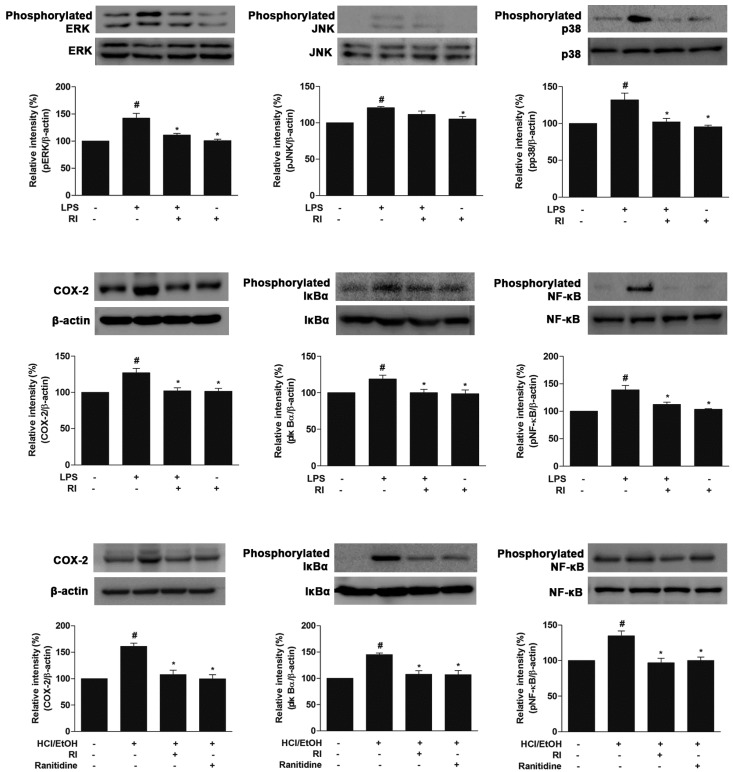
Protective role of RI on the MAPK cascades, COX-2 expression, and activation of IκBα, NF-κB in RAW 264.7 cells and gastric tissue. Here, upper and middle panels represent the MAPKs (pERK1/2, pJNK, and pp38), COX-2, IκBα and NF-κB expression in RAW 264.7 cells and the lower panel represents the COX-2, IκBα and NF-κB expression in the gastric tissue. The relative band intensity of target protein was measured as compared with β-actin. LPS-induced the phosphorylation of MAPK cascade, whereas pretreatment with the RI reduced the phosphorylation of MAPK cascade. LPS and HCl/EtOH increased the COX-2 expression, kinetic phosphorylation, and degradation of IκBα and phosphorylation of NF-κB. However, pretreatment with the RI notably decreased the COX-2 expression, IκBα phosphorylation, and degradation, NF-κB translocation as related to standard drug ranitidine. # *p* < 0.05 when compared with the control and * *p* < 0.05 when compared with LPS and HCl/EtOH. Data are expressed as mean ± SEM.

These changes have no material impact on the conclusions of our paper. The authors would like to apologize for any inconvenience caused to the readers by these changes.
